# Why combine and why neoadjuvant? Tumor immunological perspectives on chemoimmunotherapy in triple-negative breast cancer

**DOI:** 10.1007/s12282-025-01707-5

**Published:** 2025-05-06

**Authors:** Kazuhiro Kakimi, Tomoharu Sugie

**Affiliations:** 1 Department of Immunology, Kindai University Faculty of Medicine, 377-2 Onohigashi, Osakasayama, Osaka, 589-8511 Japan; 2 Chemotherapy Center, Kansai Medical University Kori Hospital, 8-45 Korihondori, Neyagawa, Osaka, 572-8551 Japan

**Keywords:** Triple-negative breast cancer (TNBC), Immune checkpoint inhibitors (ICIs), Tumor microenvironment (TME), T cell exhaustion, Epigenetic scar, Neoadjuvant and adjuvant immunotherapy

## Abstract

Triple-negative breast cancer (TNBC) is an aggressive subtype characterized by limited targeted therapies and high recurrence rates. While immune checkpoint inhibitors (ICIs) have shown promise, their efficacy as monotherapy is limited. Clinically, ICIs demonstrate significant benefit primarily when combined with chemotherapy, particularly in the neoadjuvant setting for early-stage TNBC, which yields superior outcomes compared to adjuvant therapy. This review elucidates the tumor immunological principles underlying these observations. We discussed how the suppressive tumor microenvironment (TME), progressive T cell exhaustion, and associated epigenetic scarring constrain ICI monotherapy effectiveness. Crucially, we highlight the immunological advantages of the neoadjuvant approach: the presence of the primary tumor provides abundant antigens, and intact tumor-draining lymph nodes (TDLNs) act as critical sites for ICI-mediated priming and expansion of naïve and precursor exhausted T cells. This robust activation within TDLNs enhances systemic anti-tumor immunity and expands the T cell repertoire, a process less effectively achieved in the adjuvant setting after tumor resection. These mechanisms provide a strong rationale for the improved pathological complete response (pCR) rates and event-free survival observed with neoadjuvant chemoimmunotherapy, as demonstrated in trials like KEYNOTE-522. We further explore the implications for adjuvant therapy decisions based on treatment response, the challenges of ICI resistance, the need for predictive biomarkers, management of immune-related adverse events (irAEs), and future therapeutic directions. Understanding the dynamic interplay between chemotherapy, ICIs, T cells, and the TME, particularly the role of TDLNs in the neoadjuvant context, is essential for optimizing immunotherapy strategies and improving outcomes for patients with TNBC.

## Introduction

Triple-negative breast cancer (TNBC) is an aggressive subtype lacking targeted therapies due to the absence of estrogen receptor (ER), progesterone receptor, and human epidermal growth factor receptor 2 (HER2) expression [1]. Accounting for approximately 15–20% of all breast cancers, TNBC is characterized by early onset, high recurrence risk, and limited therapeutic options [2]. Recent advancements in immunotherapy, particularly immune checkpoint inhibitors (ICIs), have shown promise in TNBC treatment [1, 3].

However, the efficacy of ICI monotherapy remains limited. For instance, KEYNOTE-086 trial (cohort A) evaluating pembrolizumab monotherapy in advanced TNBC showed an objective response rate (ORR) of only 5.3% [4]. Moreover, KEYNOTE-119 trial showed no significant improvement in overall survival (OS) compared to chemotherapy [5]. Consequently, ICI monotherapy is not established as standard treatment for advanced TNBC. Currently, ICIs are approved only in combination with chemotherapy, where clinical benefit has been demonstrated. In contrast, in early-stage TNBC, neoadjuvant (preoperative) ICI-based therapy—such as in KEYNOTE-522 and IMpassion031 trials—has gained attention due to superior clinical efficacy compared to adjuvant (postoperative) therapy [6–9]. Notably, only a subset of patients responds to ICI-based therapies, highlighting the need for predictive biomarkers and a deeper understanding of resistance mechanisms.

This review elucidates, from a tumor immunological perspective, the mechanisms underlying limited ICI monotherapy efficacy in TNBC, the rationale for combination strategies with chemotherapy, and factors contributing to the enhanced effectiveness of neoadjuvant ICI-based regimens, drawing on insights from basic and clinical research.

## Fundamentals of tumor immunology

### What is cancer immunotherapy?

Cancer immunotherapy harnesses the patient's immune system to attack cancer cells. Unlike conventional therapies, immunotherapy aims to eliminate cancer cells by activating immune cells, particularly T cells, that recognize and kill tumor cells [10]. Various modalities exist, including ICIs, chimeric antigen receptor (CAR)-T cell therapy, T cell receptor (TCR)-T cell therapy, cancer vaccines, and adjuvant therapies. These approaches rely on the effector function of T cells recognizing and targeting tumors[11–14]. Recent research has expanded beyond activating T cells to include strategies involving breaking immune tolerance, remodeling the tumor microenvironment (TME), activating innate immune cells (e.g., natural killer [NK] cells, macrophages, dendritic cells [DCs]), and normalizing tumor vasculature to promote immune cell infiltration [15].

### Immune checkpoint inhibitors and tumor-specific T cells

ICIs restore T cell anti-tumor activity by blocking immune checkpoint molecules suppressing T cell activation [10]. The presence of tumor-specific T cells is essential for ICI effectiveness [10, 11]. These T cells recognize cancer antigens (e.g., neoantigens, cancer-testis antigens, differentiation antigens) and can specifically attack cancer cells [11]. Neoantigens, novel antigens from cancer-specific mutations, can potentially elicit personalized immune responses. ICIs enhance anti-tumor effects by restoring function and promoting proliferation of these tumor-specific T cells. Combinations of ICIs with neoantigen vaccines or adjuvant therapies are under investigation to improve ICI efficacy [16, 17].

### T cell function and differentiation

T cells are classified into subsets based on function and differentiation state, including naïve T cells, effector T cells (Teff), memory T cells (Tm), exhausted T cells (Tex), and tissue-resident memory T (Trm) cells [18–20].

- Naïve T cells: T cells yet to encounter antigen. Reside in secondary lymphoid organs (lymph nodes) awaiting stimulation from antigen-presenting cells (APCs).
- Teff: T cells activated by antigen recognition, possessing the ability to attack tumor cells. Includes cytotoxic T lymphocytes and helper T cells.
- Tm: T cells that previously responded to antigen. Mediate long-term immunological memory and mount rapid, robust responses upon re-exposure.
- Trm-like cells: Trm cells are memory T cells residing in tissues providing local immune protection. In tumors, a similar population expressing Trm markers (CD69, CD103) exists. These "Trm-like cells" face chronic tumor antigen exposure and often express exhaustion markers like programmed cell death (PD)-1 and T-cell immunoglobulin and mucin domain (TIM)-3, indicating potential functional impairment.
- Tex: T cells found in chronic viral infections and tumors. Become dysfunctional due to persistent antigen stimulation and suppressive TME factors. Highly express immune checkpoints like PD-1.

Each subset has distinct characteristics and roles, functioning coordinately in anti-tumor immune responses.

### PD-1 expression and function

PD-1 is an immune checkpoint molecule on activated T cells. Binding PD-1 to its ligands, PD-L1 and PD-L2, inhibits TCR signaling, regulating excessive T cell activation [21]. Excessive activation of T cells can induce activation-induced cell death (AICD). PD-1 is necessary for appropriate immune responses by modulating TCR signaling of antigen-stimulated T cells, thereby suppressing AICD. PD-1 signaling also suppresses activation of self-reactive T cells, preventing autoimmune diseases [21]. Therefore, PD-1 is not only an exhaustion marker or inhibitory molecule but also a key regulator fine-tuning T cell activation, maintaining immune balance, and preventing excessive responses.

The association between PD-L1 expression and ICI therapeutic effect varies by cancer type. In non-small cell lung cancer and malignant melanoma, higher PD-L1 expression is associated with higher ICI efficacy [22]. Conversely, in breast cancer, especially TNBC, the PD-L1/ICI effect association is inconsistent; ICIs can be effective regardless of PD-L1 expression in early-stage TNBC [6]. Tumor cell PD-L1 expression can be induced by interferon (IFN)-γ, suggesting tumor-infiltrating lymphocyte (TIL) presence [21]. However, PD-L1 expression is controlled by complex pathways (gene mutations, transcription, mRNA stability, oncogenes, protein stability) and does not necessarily correlate with anti-tumor CD8^+^ T cell responses [23]. Furthermore, PD-L1 expression in the TME changes dynamically, influenced by intrinsic factors and external stimuli. Thus, predicting ICI therapeutic effect solely on pre-treatment PD-L1 expression is difficult. Comprehensive evaluation combining PD-L1 with additional biomarkers and immune profiling is required to select suitable patients for ICI [22].

### T cell exhaustion and TME

**Fig. 1:**
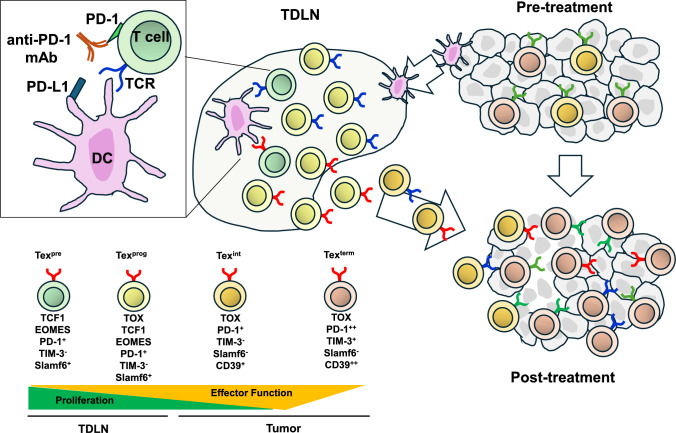
Progressive differentiation of exhausted T cell lineage subsets. Naïve CD8^+^ T cells gradually become exhausted within TME. This process progresses from stem cell-like Tex^pre^ to Tex^prog^, Tex^int^, and Tex^term^. As exhaustion progresses, T cell function and proliferative capacity gradually decline, and eventually, excessive stimulation can lead to cell death. In particular, proliferative capacity and anti-tumor effector activity are inversely related: high proliferative capacity is associated with low effector activity, while high effector activity corresponds to reduced proliferative capacity. The effect of anti-PD-(L)1 antibody therapy is exerted by inhibiting the interaction between DCs and precursor exhausted CD8^+^ T cells in TDLNs. TCR, T cell receptor; DC, dendritic cell; PD-(L)1, programmed cell death- (ligand)1; TCF-1,T-cell factor 1; TOX; thymocyte selection-associated high mobility group box; EOMES, eomesodermin; TIM-3, T cell immunoglobulin and mucin domain-containing-3; slamf6, signaling lymphocyte activation molecule family member 6; TME, tumor microenvironment; Tex^pre^, precursor exhausted T cells; Tex^pro^, progenitor exhausted T cells; Tex^int^, intermediate exhausted T cells; Tex^term^, terminally exhausted T cells; TDLNs, tumor-draining lymph nodes

The TME comprises cancer cells, immune cells, blood vessels, fibroblasts, extracellular matrix, and other components, significantly influencing immune responses [24]. Within the tumor, tumor-specific T cells face persistent antigen stimulation and suppressive factors (tumor growth factor [TGF]-β, IL-10, vascular endothelial growth factor [VEGF], hypoxia, nutrient deprivation), leading to progressive dysfunction, termed T cell exhaustion [19]. T cell dysfunction involves stepwise differentiation from precursor-exhausted T (Tex^pre^) cells to terminally exhausted T (Tex^term^) cells [25, 26] (Fig. 1 and Table 1). Progenitor-exhausted T (Tex^prog^) cells (TCF-1^+^PD-1^int^TIM3^−^Slamf6^+^) are abundant in lymph nodes, have self-renewal capacity but weak anti-tumor effector activity. Activated T cells acquire effector function, differentiate into intermediate exhausted T (Tex^int^) cells, and infiltrate the tumor. Within the tumor, effector activity eventually declines, and Tex^term^ cells in the terminal differentiation stage with low self-renewal capacity are generated [25].

**Table 1:** Characteristics of exhausted CD8⁺ T cell subsets

Feature	Precursor Exhausted T (Tex^pre^)	Progenitor Exhausted T (Tex^prog^)	Intermediate Exhausted T (Tex^int^)	Terminally Exhausted T (Tex^term^)
Key surface markers	PD-1^+^, TIM-3^-^, Slamf6^+^	PD-1^+^, TIM-3⁻, Slamf6^+^	PD-1^++^TIM-3^-^, Slamf6^-^, CD39^+^,	PD-1^++^, TIM-3⁺, Slamf6^-^, CD39^++^
Transcription factor profile	TCF1, EOMES,	TCF1,TOX, EOMES	TOX, Loss of TCF1	High TOX,
Functional characteristics	Early exhaustion; retains differentiation potential	Stem-like; self-renewing and gives rise to downstream subsets	Transitional; declining effector function	Fixed phenotype; poor cytokine production; high inhibitory receptor expression
Proliferative capacity	Moderate	High	Low	Very low
Responsiveness to ICB therapy	Responsive	Highly responsive	Partially responsive	Non-responsive
Remarks	Transitional from naïve/memory state	Key subset for antitumor immunity and therapeutic efficacy	May migrate from lymph nodes or blood to TME	Terminally differentiated; unlikely to be reprogrammed

TCF1, T cell factor 1; PD-1, programmed cell death-1; EOMES, eomesodermin; TIM-3, T-cell immunoglobulin and mucin domain 3; slamf6, signaling lymphocyte activation molecule family member 6; TOX; thymocyte selection-associated high mobility group box; TME, tumor microenvironment

### Mechanism of action of ICIs and the importance of tumor draining lymph nodes

ICIs exert anti-tumor effects by relieving TME immunosuppression and promoting tumor-specific T cell activation and proliferation. By blocking checkpoints like PD-1 and cytotoxic T lymphocyte antigen (CTLA)-4, ICIs restore Tex cell anti-tumor activity. This effect is important in tumor tissue and lymph nodes [19, 25]. CTLA-4 inhibitors primarily enhance T cell activation by promoting APC-T cell interactions in lymph nodes. In contrast, anti-PD-(L)1 antibodies, initially thought to restore exhausted T cell function within the TME, are now known to largely mediate their effect by disrupting interactions between DCs and CD8^+^ Tex^pre^ cells in TDLNs. PD-1 inhibitors promote Tex^pre^ and Tex^prog^ activation and proliferation in lymph nodes, enhancing T cell infiltration into the tumor [27]. Furthermore, Tex^prog^ acquires anti-tumor effector activity upon tumor infiltration and differentiation to Tex^int^. Tex^pre^ and Tex^prog^ are essential for amplifying tumor-specific T cells [25, 26]. Lymph nodes play a crucial role in initiating and regulating immune responses by serving as sites for naïve T cell activation and Tex^pre^/Tex^prog^ proliferation. This immunological function provides a strong rationale for the superiority of neoadjuvant therapy. Nevertheless, lymph node dissection can reduce the number of immune cells present, potentially impairing the efficacy of immunotherapy [28]. To mitigate this risk, less invasive procedures such as sentinel lymph node biopsy are recommended [29]. Furthermore, both the extent of lymph node metastasis and the immune cell composition within the nodes may influence the therapeutic outcomes of immune checkpoint inhibitors (ICIs) [30].

### Epigenetic scarring in T cell exhaustion and its impact on immunotherapy

**Fig. 2:**
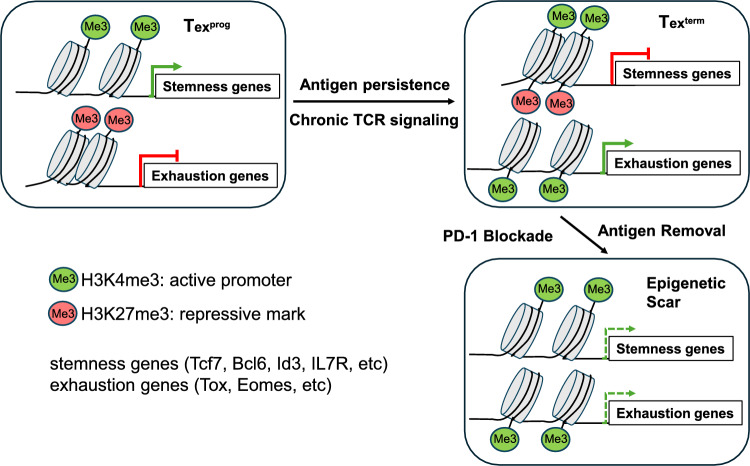
Epigenetic regulation of T cell exhaustion and epigenetic scarring after antigen removal. Persistent TCR stimulation is considered a major factor in T cell exhaustion. Dynamic changes occur in the epigenome during the process of differentiation of precursor CD8^+^ T cells into an exhausted state. Specifically, chromatin accessibility increases in effector genes and exhaustion-related genes, while chromatin accessibility decreases in genes related to stem cell properties. These epigenetic changes are mediated by changes in DNA methylation and histone modifications. When chronic antigen stimulation is removed, Tex rapidly convert to a less exhausted, memory-like phenotype. Molecular profiling of these recovered Tex cells confirmed that chromatin accessibility of stem cell-related genes such as IL-7R was partially restored. However, epigenetically accessible "scars" remain in exhaustion-related regions, which may prevent recovered Tex cells from being completely reprogrammed into functional memory T cells. TCR, T cell receptor; Tex^pro^, progenitor exhausted T cells; Tex^term^, terminally exhausted T cells; H3, histone H3; me3, trimethylation; Tcf7, T cell factor 7; Bcl6, B-cell/CLL lymphoma 6; Id3, inhibitor differentiation3; 1L-7R, interleukine-7 receptor

While ICIs target inhibitory receptors like PD-1 to promote Tex cell functional recovery, recent studies suggest epigenetic fixation limits this recovery. Epigenetic scarring, including accumulation of repressive histone modifications (e.g., H3K27me3), altered DNA methylation patterns, and decreased chromatin accessibility in effector gene regions is a contributing factor [31] (Fig. 2). These changes cause Tex cells to lose activation responsiveness, making complete functional recovery difficult even after checkpoint inhibition. Whether this epigenetic scar is reversible upon removal of persistent antigen stimulation or is irreversible remains unclear. Studies of antiviral therapy for hepatitis C virus reported that Tex function does not fully recover even after virus elimination, and epigenetic scars remain [32, 33]. Similarly, chronic stimulation by tumor antigens has been shown to induce T cell exhaustion, which is associated with epigenetic changes, particularly alterations in chromatin accessibility. Notably, these epigenetic modifications persist even after immune checkpoint blockade therapy, limiting the full functional recovery of exhausted T cells. ATAC-seq analysis demonstrated that the epigenetic profile of exhausted T cells remains largely unchanged following PD-1 blockade, resulting in only partial restoration of function [34]. These findings suggest the significance of postoperative adjuvant therapy. Even if pCR is achieved with neoadjuvant therapy, Tex function may not be fully restored; appropriate postoperative ICI therapy might be required to prevent recurrence and maintain long-term immune surveillance. Other strategies to restore Tex function, such as therapies targeting epigenetic reprogramming, should also be considered [35]. Future research needs to clarify the reversibility of Tex cell epigenetic fixation and establish optimal treatment strategies based on it.

## Immunotherapy for TNBC

### Immunological features of TNBC

In normal breast tissue, immune cells are generally sparse. However, in breast cancer, TNBC tends to have higher TIL density compared to other subtypes [36]. Abundant TILs generally indicate that Teff, Tm, and Trm-like cells are actively present in the TME, and the immune system is actively recognizing and attempting to fight the tumor. Breast cancer has moderate tumor mutation burden (TMB) compared to other cancers, but TNBC often exhibits higher TMB than other subtypes [37]. High TMB contributes to neoantigen generation, recognizable by the immune system, thereby increasing the likelihood of an immune response in TNBC. As activated TILs release cytokines, particularly IFN-γ, PD-L1 expression is higher in TNBC, leading to higher ICI response rates.

In contrast, ER-positive breast cancer typically has lower TIL levels, less PD-L1 positivity, and lower TMB. Recent studies reported associations between ER signaling and immune suppression. For example, ERα signaling in tumor-associated macrophages contributed to an immune-suppressive TME state by promoting CD8^+^ T cell dysfunction and exhaustion [38]. Another study reported that hormone signaling can suppress the immune system by decreasing immune cell infiltration [39]. As TNBC lacks hormone-driven immune suppression, the immune system can play a more active role in tumor recognition and attack. For these reasons, breast cancer immunotherapy has primarily focused on and developed for TNBC.

### Metastatic TNBC

Advances in tumor immunology and molecular targeted therapy have dramatically evolved metastatic TNBC treatment strategy. Metastatic TNBC treatment requires a multifaceted approach tailored to patient's tumor biology and history. Conventional chemotherapies aim for rapid tumor shrinkage, but resistance and side effects necessitate balancing long-term sustainability with quality of life. For the second line and beyond, poly(ADP-ribose) polymerase (PARP) inhibitors (e.g., olaparib, talazoparib) exploit DNA repair deficiencies to effectively control tumor growth, extending progression-free survival (PFS) and representing personalized treatment in *BRCA1/2*-mutated TNBC. Antibody drug conjugates (ADCs) link cytotoxic drugs to tumor-targeting antibodies such as trastuzumab deruxtecan (HER2-positive or HER2-low) [40]and sacituzumab govitecan (TNBC) [41] improved objective response rates (ORR) and progression-free survival (PFS) compared to chemotherapy.

As the first-line treatment, ICIs (e.g., pembrolizumab, atezolizumab) combined with chemotherapy achieve excellent clinical outcomes, particularly in PD-L1-positive TNBC. Both KEYNOTE‑355 (using pembrolizumab with one of three chemotherapy regimens: nab‑paclitaxel, paclitaxel, or gemcitabine plus carboplatin) and IMpassion130 (using atezolizumab with nab‑paclitaxel) reported significant improvements in progression‑free survival (PFS). [42, 43]. Pembrolizumab also provided significant OS benefit [44]. Based on the results of these clinical trials, pembrolizumab (or atezolizumab if available) with chemotherapy is now the standard of care for PD-L1-positive metastatic TNBC.

In contrast, IMpassion131 (using atezolizumab with paclitaxel) showed no significant benefits [45]. Reasons for differing results remain unclear but likely stem from interconnected factors. Subtle differences in patient clinical characteristics (age, prior treatment, PD-L1 status) might exist. Prior treatments/conditions may affect the immune system/TME, influencing atezolizumab efficacy. Chemotherapy agent choice may be critical. Nab-paclitaxel (IMpassion130) is albumin-bound, not requiring corticosteroid premedication, whereas paclitaxel (IMpassion131) does. Corticosteroids, necessary for paclitaxel hypersensitivity prevention, can suppress immune function, potentially diminishing PD-L1 blockade's essential immune activation. Moreover, nab-paclitaxel reportedly exerts a more favorable immunomodulatory profile (enhancing antigen presentation, preserving T-cell function, boosting mast cells), leading to more robust anti-tumor responses [46]. Differences in drug delivery/penetration between paclitaxel and nab-paclitaxel may also cause distinct TME effects.

### Early-stage TNBC

Immunotherapy in the neoadjuvant setting is now the standard of care for early-stage TNBC. The KEYNOTE-522 trial examined neoadjuvant pembrolizumab plus chemotherapy (including platinum) followed by adjuvant pembrolizumab in high-risk early TNBC. Pembrolizumab significantly improved pathological complete response (pCR) rate, prolonged event-free survival (EFS) and OS. In contrast, in IMpassion031, adding atezolizumab to chemotherapy significantly improved pCR and showed a trend toward better prognosis, although the prognosis improvement was not statistically significant [9]. In KEYNOTE-522, pembrolizumab was given both before and after surgery, which helped sustain immune activation whereas IMpassion031 used atezolizumab only as neoadjuvant therapy, possibly limiting the long-term immune benefit. Pembrolizumab, a PD-1 inhibitor, blocks immune suppression more broadly and can activate a more robust, sustained antitumor response. Atezolizumab, a PD-L1 inhibitor, might provide only a transient boost in immunity. Differences in the chemotherapy regimens (KEYNOTE522: carboplatin/paclitaxel then anthracycline/cyclophosphamide [AC]; IMpassion031: nab-paclitaxel then AC) may influence immune activation and its synergy with immunotherapy. These differences likely explain why KEYNOTE-522 showed improvements in both short- and long-term outcomes, while IMpassion031 only showed a significant pCR improvement.

In the ALEXANDRA/IMpassion030 trial, early-stage TNBC patients received adjuvant chemotherapy (paclitaxel then dose-dense AC) with or without atezolizumab, then maintenance atezolizumab. However, atezolizumab did not enhance the effect of adjuvant chemotherapy [47]. IMpassion030 used adjuvant (postoperative) immunotherapy only, whereas KEYNOTE‐522 and IMpassion031 applied the checkpoint inhibitors in a neoadjuvant setting (or both neoadjuvant and adjuvant in KEYNOTE-522). From an immunological perspective, the timing of immunotherapy appears critical. In neoadjuvant settings, the presence of the primary tumor provides a continuous source of tumor antigens, enabling effective priming of naïve T cells through DC presentation. This not only reactivates pre-existing Tex—many of which express PD-1—but also promotes the generation of new T cell clones, thereby broadening the T cell repertoire and enhancing anti-tumor responses [48, 49]. These immunological events occur both within the tumor and TDLNs, maximizing the immune activation window. In contrast, adjuvant immunotherapy is administered after tumor resection, when the source of tumor antigens is largely eliminated. This limits new T cell priming and may reduce the overall efficacy of ICIs. Accordingly, neoadjuvant immunotherapy may offer superior anti-tumor effects in early-stage TNBC by harnessing a more diverse and robust T cell response [50]

### The need for adjuvant Immunotherapy in pCR cases

The KEYNOTE-522 was designed to administer adjuvant pembrolizumab regardless of the response to neoadjuvant immunotherapy. As patients achieving pCR demonstrated excellent clinical outcomes, it remains to be determined whether this adjuvant treatment provides extra benefits for all patients. In GeparNuevo trial [51], while EFS improved with preoperative durvalumab and chemotherapy, no postoperative therapy was given. This suggests long-term benefits might be achievable with preoperative ICI alone. Notably, patients achieving pCR showed 100% three-year distant disease-free survival and OS. We do not clearly know if adjuvant ICI is required for patients achieving pCR.

**Fig. 3:**
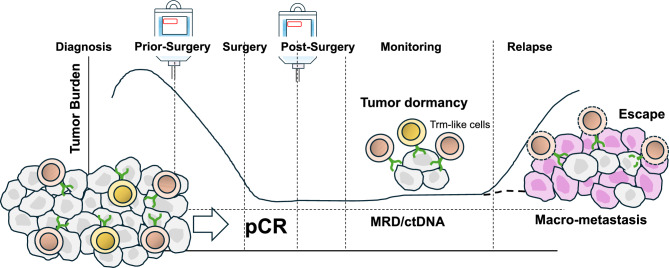
Perioperative immunotherapy and tumor dynamics. Neoadjuvant immunotherapy can efficiently activate pre-existing effector T cells and expand tumor-specific T cell clones. These activated T cells infiltrate the tumor and differentiate into tissue-resident memory-like T cells (Trm-like cells), which localize and persist within the tumor bed. During the neoadjuvant phase, such localized immune activation contributes to significant tumor shrinkage. Importantly, Trm-like cells remain stationed at the former tumor site after surgery. If residual cancer cells begin to re-grow locally, these Trm-like cells can rapidly recognize them and initiate a robust, site-specific immune response, thereby preventing local recurrence. However, even in cases where pCR is achieved, ctDNA may still be detectable, indicating the presence of MRD elsewhere in the body. In the adjuvant phase, systemic administration of immune checkpoint inhibitors (ICIs) can stimulate a broader population of memory T cells—including circulating or lymphoid-residing memory T cells—that can detect and targeting MRD throughout the body. By supporting both the maintenance of Trm-like cells in the surgical bed and enhancing systemic memory T cell responses, ICIs can help maintain tumor dormancy. Nonetheless, if this immune surveillance network is disrupted, tumor cells may escape immunological control, leading to renewed proliferation and recurrence as distant macro-metastases. Trm-like cell, resident memory-like T cells; pCR, pathological complete response; MRD, minimal residual disease; ctDNA, circulating tumor DNA

In pCR cases (tumor completely absent at surgery), tumor-specific T cell function is likely preserved, suggesting potentially limited need for adjuvant immunotherapy. However, since pCR is determined pathologically, minimal residual disease (MRD) undetectable by imaging/diagnostics cannot be entirely excluded. Although pCR is a favorable prognostic indicator, recent studies have highlighted its limitations as a surrogate marker for complete immune eradication of tumor cells. Detection of circulating tumor DNA (ctDNA) in patients with pCR suggests the presence of MRD, which may lead to future recurrence if not addressed [52]. Supporting this, ctDNA levels were significantly lower in patients who achieved pCR after neoadjuvant immunotherapy compared to non-pCR patients, yet still detectable in some cases [53]. Therefore, adjuvant immunotherapy might help eliminate systemic minimal residual disease by enhancing the activity and persistence of circulating memory T cells, potentially contributing to long-term suppression of distant recurrence. In parallel, at the surgical site, the re-activation of pre-existing Trm-like cells may play a central role in preventing local tumor relapse [54] (Fig. 3). Conversely, a cautious approach is warranted due to potential irreversible irAE risks [55, 56]. Since recurrence risk in pCR patients is low, adjuvant immunotherapy benefits may not outweigh irAE harms. Medical economics are also important; ICI is expensive, and post-surgery administration adds significant burden. Currently, based on KEYNOTE-522 protocol, maintaining adjuvant pembrolizumab after surgery is considered reasonable for TNBC patients. However, when patients achieve pCR, the treatment plan should comprehensively consider their condition, irAE risk, and economic toxicity. The OptimICE-PCR trial (NCT05812807) is planned to investigate whether adjuvant immunotherapy can be omitted after pCR. It randomizes patients achieving pCR (with ≥ 6 cycles neoadjuvant pembrolizumab) to observation or continued pembrolizumab. OptimICE-PCR results are expected to provide a definitive answer regarding adjuvant immunotherapy necessity in pCR cases.

### Adjuvant immunotherapy for residual disease after neoadjuvant immunotherapy

According to KEYNOTE-522 data, patients with residual cancer burden (RCB)-I demonstrated outcomes as favorable as RCB-0 (equivalent to pCR), suggesting minimal need for adjuvant pembrolizumab. Patients with RCB-II exhibited the greatest EFS improvement (hazard ratio [HR] 0.52) among RCB categories [57]. RCB‐II represents an intermediate-risk group in which a complete response was not achieved, yet a partial response to neoadjuvant chemo- and immunotherapy was observed. In this group, the presence of residual tumor may lead to the release of tumor antigens and an increase in immunogenicity, thereby potentially re-educating or boosting the immune response. On the other hand, RCB-III prognosis remains poor (HR 1.24), with limited benefit from adjuvant pembrolizumab. RCB‐III represents a group that shows primary resistance to neoadjuvant therapy, suggesting a high degree of resistance to both chemo- and immunotherapy. Thus, relying solely on adjuvant pembrolizumab may be insufficient, and alternative treatment strategies are needed. Indeed, few physicians consider pembrolizumab alone sufficient for non-pCR patients; administration of capecitabine or olaparib (for *BRCA*-mutated TNBC) is becoming widespread. Furthermore, ADCs showing excellent effects against breast cancer have been developed. OptimICE-RD trial (sacituzumab govitecan + pembrolizumab) [58] and TROPION-Breast03 trial (datopotamab deruxtecan + durvalumab) [59] are currently underway. These trial results are expected to further optimize the adjuvant treatment strategy for TNBC [60].

## Challenges and countermeasures in immunotherapy

### Immune-related adverse events

ICIs can cause irAEs due to excessive immune system activation [55, 56]. irAEs can occur in various organs; severe cases may necessitate treatment discontinuation. For irAEs, early detection and treatment by a multidisciplinary team (oncology, specialists, nurses, pharmacists) is important. Patient education encouraging self-management is also crucial for early detection. Compliance with irAE diagnosis and treatment guidelines [61, 62] is important for appropriate management. Depending on severity, steroids or immunosuppressants may be necessary. Research is progressing on biomarkers predicting irAE risk and treatments preventing irAE [63].

### Biomarker discovery

Discovering biomarkers predicting immunotherapy efficacy is an important challenge [64]. TILs are considered promising predictors. As mentioned, TIL density and constituent immune cell types may affect ICI efficacy. T cell differentiation status and functional analysis are also important. Recent advances in single-cell and spatial profiling technologies led to proposals of novel biomarkers based on intratumoral immune responses. Bassez et al. conducted comprehensive single-cell transcriptome, TCR repertoire, and proteome analysis in tumor samples pre/post-neoadjuvant anti-PD-1 therapy (ER-positive or TNBC) [65]. Findings revealed clonal expansion of tumor PD-1-expressing T cells post-therapy. Presence of specific immune populations (PD-L1^+^ immunoregulatory DCs, CCR2^+^ or MMP9^+^ macrophages, MHC class I/II^+^ cancer cells) positively correlated with T cell expansion. Conversely, undifferentiated precursor/memory T cells (TCF-1^+^, GZMK^+^) and immunosuppressive macrophages (CX3CR1^+^, C3^+^) negatively correlated. Notably, pre-treatment PD-1 expression levels, Tex cell abundance, and T cell clonality/richness predicted T cell expansion more accurately than traditional biomarkers like TIL scores or TMB. Additionally, diverse NK cell subsets, intratumoral epithelial heterogeneity, and intercellular interactions were suggested to influence ICI therapeutic response [66]. While single-cell analyses provide detailed functional insights, spatial transcriptomics enables integrating spatial context with gene expression, offering more comprehensive TME understanding. Using high-resolution spatial transcriptomics, Wang et al. characterized complex TNBC intratumoral heterogeneity, identifying nine distinct spatial archetypes reflecting tumor/stromal spatial organization and tertiary lymphoid structure molecular features [67]. These findings hold promise for patient stratification, prognostication, and developing personalized therapeutic strategies, including immunotherapy. Taken together, these findings underscore the importance of comprehensive, high-resolution breast cancer TME analyses—capturing cellular composition and spatial organization—for identifying novel predictive biomarkers and therapeutic targets.

### Resistance to ICIs and future perspectives

Although a decade has passed since ICI introduction, a considerable number of patients still exhibit resistance [68]. To further optimize immunotherapy, unraveling complex TME immunosuppressive mechanisms and promoting comprehensive research from multiple perspectives is essential. Mechanisms underlying ICI resistance are summarized in Table 2 (due to space constraints) In addition, several excellent reviews have comprehensively discussed the mechanisms of resistance to ICIs in TNBC. Readers interested in more detailed information are encouraged to refer to these publications.

**Table 2:** Mechanisms of resistance to ICIs in TNBC

Mechanism	Description	Reference
Immunosuppressive TME	CAF and TAM-derived TGF-β, IL-10 promote an immunosuppressive TME that impairs T cell activation	Said et al. [73]
Heterogeneous and dynamic PD-L1 expression	PD-L1 expression is spatially and temporally variable, influenced by inflammation and sampling site Assay variability and expression dynamics limit the predictive power of PD-L1 testing	Li et al. [74]
Low TMB and MSI	TNBC has intermediate TMB and low MSI frequency, limiting neoantigen availability and immune recognition	O'Meara et al. [75], Chalmers et al. [76]
Defective antigen presentation (MHC-I loss/heterogeneity)	Loss or downregulation of MHC-I disrupts antigen presentation and contributes to ICI resistance	Taylor et al. [77]
Insufficient T cell infiltration (immune desert/exclusion)	T cells may be absent or trapped in stroma, preventing effective tumor infiltration and immune attack	So et al. [78]
T cell exhaustion (expression of multiple checkpoints)	Chronic antigen exposure leads to exhausted T cells expressing PD-1, LAG-3, TIM-3, TIGIT, etc	Serrano Garcia et al. [79]
Accumulation of suppressive immune cells (Treg, MDSC, TAM)	Treg, MDSC, and M2-TAM inhibit effector T cells via cytokines, nutrient depletion, ROS, and IDO	Serrano Garcia et al. [79]
Alternative immune checkpoints (LAG-3, VISTA, B7-H4)	Non-PD-1 checkpoints can compensate, reducing the efficacy of PD-1/PD-L1 blockade alone	Serrano Garcia et al. [79]
Defective IFN signaling (JAK/STAT mutations)	Mutations in IFN pathways hinder MHC-I induction and anti-tumor immunity, driving resistance	Taylor et al. [77]
Immunosuppressive tumor metabolism (lactate, adenosine)	Tumor-produced lactate and adenosine suppress T cells through metabolic stress and A2A signaling	Serrano Garcia et al. [79]
Disrupted chemokine expression (low CXCL9/10, high CXCL1/2)	Low CXCL9/10 limits effector cell trafficking; high CXCL1/2 attracts suppressive cells like MDSCs	Serrano Garcia et al. [79]

ICI, immune checkpoint inhibitor; TNBC, triple-negative breast cancer; TME, tumor microenvironment; CAF, cancer-associated fibroblast; TAM, tumor-associated macrophage; TGF-β tumor growth factor-β; IL-10, interleukin-10; PD-(L)1, programmed cell death-(ligand) 1; TMB, tumor mutation burden; MSI, microsatellite instability; MHC-I, major histocompatibility complex- class I; LAG-3, lymphocyte activation gene-3; TIM-3, T-cell immunoglobulin and mucin domain-3; TIGIT, T cell immunoreceptor with Ig and ITIM domains; MDSC, myeloid-derived suppressor cell; ROS, reactive oxygen species; IDO, indoleamine 2,3 dioxygenase; VISTA, V-domain Ig suppressor of T cell activation; IFN, interferon; JAK/STAT, Janus kinase/signal transducers and activators of transcription; CXCL, chemokine (C-X-C motif) ligand

Future challenges in TNBC immunotherapy involve addressing current treatment limitations and developing more effective, personalized approaches (Table 3) to overcome ICI resistance. While ICIs show promise, a significant proportion do not respond to monotherapy, highlighting the urgent need for novel strategies. Development of new drugs for ICI combination is active, including ADCs, anti-angiogenic agents, PARP inhibitors, and phosphatidylinositol-3 kinase (PI3K) inhibitors. In TNBC, overexpression of LDHA leads to excessive lactate accumulation and acidification of the tumor microenvironment, which suppresses antitumor immune responses and contributes to resistance to ICIs. These findings highlight the need for therapeutic strategies that incorporate metabolic reprogramming to overcome ICI resistance in TNBC[69]. EZH2, a histone methyltransferase and core component of the Polycomb Repressive Complex 2 (PRC2), is a key epigenetic regulator frequently overexpressed in invasive breast cancer and closely associated with tumor progression and metastasis [70]. By repressing immune-related genes such as CXCL9/CXCL10**,** MHC class I molecules, and interferon-stimulated genes**,** EZH2 contributes to immune evasion through reduced T cell recruitment and impaired antigen presentation [71]. It also shapes the tumor microenvironment by promoting regulatory T cell (Treg) activity and myeloid-derived suppressor cell (MDSC) development and alters tumor metabolism by suppressing lactate and IDO expression, thereby preserving T cell function. Given these immunosuppressive roles, EZH2 inhibition is being explored in combination with immune checkpoint inhibitors to enhance antitumor immunity and overcome therapeutic resistance in breast cancer [72].

**Table 3:** Future challenges to be addressed

**Basic research**
Elucidation and overcoming of resistance mechanisms
Deeper understanding of the immunological role of lymph nodes
Establishment of therapeutic strategies considering the diversity of TME
**Optimization of treatment**
Establishment of optimal combination strategies of ICI in preoperative and postoperative settings
Development of systems for early detection and management of irAEs
Establishment of treatment strategies that consider patients' Quality of Life (QOL)
Identification of predictive biomarkers for ICI efficacy and realization of personalized medicine
Promotion of immune cell infiltration through normalization of tumor vasculature
Improvement of immune response through regulation of gut microbiota and nutritional metabolism
**Development of novel therapies**
Development of therapies targeting novel immune checkpoint molecules
Clinical application of T-cell transfer therapies (CAR-T cell therapy, TCR-T cell therapy)
Development and clinical application of cancer vaccines
Clinical application of adjuvant therapies (e.g., TLR agonists, STING agonists)
Development of immunotherapies that activate innate immune cells (e.g., NK cells, macrophages, DCs)

TME, tumor microenvironment; ICI, immune checkpoint inhibitor; irAE, immune-related adverse event; CAR-T, chimeric antigen cell receptor T cell; TCR, T cell receptor; TLR, Toll-like receptor; STING, stimulator of interferon genes; NK, natural killer, DCs, dendritic cells

Combining ICIs with cell-based therapies (CAR-T, TCR-T) is expected to further enhance efficacy. Other promising approaches include combination with radiation therapy and modulating the immune environment via gut microbiota regulation. In basic research, elucidating ICI resistance mechanisms, gaining deeper insights into lymph node immunological roles, and developing therapeutic strategies accounting for TME heterogeneity are essential. To optimize treatment, efforts should focus on identifying predictive biomarkers, managing irAEs, alleviating cost burden, and maintaining patient quality of life. For tumors with low TMB or impaired antigen presentation, CAR-T cell therapies targeting tumor-specific surface antigens represent a promising alternative. Moreover, converting immunologically "cold" tumors into "hot" tumors using cancer vaccines and adjuvant therapies (TLR agonists, STING agonists) is an active investigation area. Ultimately, integrating these diverse therapeutic strategies and building robust clinical evidence will be essential for improving outcomes and achieving personalized TNBC treatment.

## Conclusion

Immunotherapy with ICIs has revolutionized TNBC treatment, showing promising efficacy in neoadjuvant and metastatic settings. Understanding T cell exhaustion, TME, and resistance mechanisms, such as epigenetic scarring, is crucial for optimizing treatment strategies. Notably, T cell activation and priming within tumor-draining lymph nodes represent a critical step in initiating and sustaining effective anti-tumor immune responses. T cells undergo dynamic activation and differentiation migrating across distinct anatomical sites, shaping their effector functions and memory potential. Future research should focus on overcoming resistance, minimizing irAEs, and refining personalized therapies, including biomarker-driven immunotherapy, to enhance the durability of anti-tumor responses in patients with TNBC.

## Data Availability

Not applicable.
